# Supplementary material for: Transcriptomic Insights into Mechanisms of Early Seed Maturation in the Garden Pea (Pisum sativum L.)
Source: Cells. 2020 Mar 23;9(3):779. doi: 10.3390/cells9030779 (PMC7140803; doi:10.3390/cells9030779)

Sprint−2(10) vs Sprint−

Global Summary

%DE = 0.81

genes with fdr < 0.2 = 457 (247 + / 210 −)

genes with fdr < 0.1 = 407 (227 + / 180 −)

genes with fdr < 0.05 = 366 (207 + / 159 −)

genes with fdr < 0.01 = 238 (144 + / 94 −)

genes in genesets = 0

<FC> = 0

<t−score> = −121.11

<p−value> = 0.01

<fdr> = 0.19

Portrait

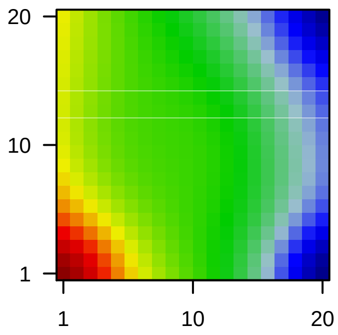

Top 100 DE genes

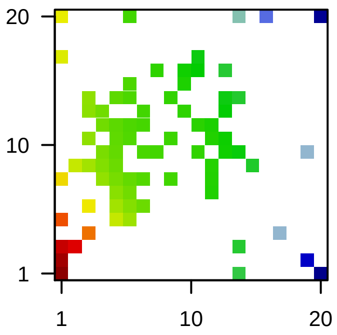

Global Genelist

Rank	ID	log(FC)	p−value	fdr	Metagene	Description
1	TRINITY_DN1	−11.1	7e−15	1e−09	20 x 20	
2	TRINITY_DN1	−19.01	1e−11	2e−08	20 x 1	
3	TRINITY_DN2	−5.88	2e−10	1e−05	20 x 1	
4	TRINITY_DN3	0.05	2e−07	1e−05	5 x 9	
5	TRINITY_DN2	0.12	2e−07	2e−05	6 x 5	
6	comp39525_c	−0.16	7e−07	2e−05	6 x 8	
7	TRINITY_DN6	−0.1	9e−07	2e−05	6 x 20	
8	TRINITY_DN4	5.5	1e−06	2e−05	1 x 1	
9	TRINITY_DN1	0.02	1e−06	2e−05	7 x 13	
10	TRINITY_DN4	0.06	1e−06	2e−05	6 x 7	
11	TRINITY_DN4	−0.61	1e−06	2e−05	14 x 1	
12	TRINITY_DN1	0.11	1e−06	2e−05	5 x 6	
13	TRINITY_DN1	0.39	2e−06	2e−05	1 x 20	
14	TRINITY_DN3	0.07	2e−06	2e−05	5 x 8	
15	TRINITY_DN1	0.04	2e−06	8e−05	5 x 12	
16	TRINITY_DN8	0.45	3e−06	8e−05	1 x 17	
17	TRINITY_DN8	0.25	4e−06	8e−05	1 x 8	
18	TRINITY_DN9	0.07	4e−06	8e−05	4 x 13	
19	TRINITY_DN2	0.62	5e−06	8e−05	1 x 2	
20	TRINITY_DN3	−0.96	5e−06	1e−04	19 x 2	

p−values

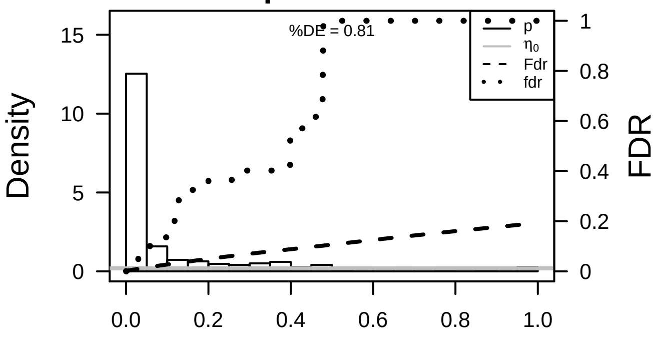

Supplement: Supplementary file 1 [file cells-09-00779-s001.zip › Supplementary materials/FileS3.zip!/FileS2/Hormones - Results/Summary Sheets - Differences/Reports/Sprint.2.10..vs.Sprint.2.20..pdf]
